# Successful Anti-CLL1 CAR T-Cell Therapy in Secondary Acute Myeloid Leukemia

**DOI:** 10.3389/fonc.2020.00685

**Published:** 2020-05-27

**Authors:** Hui Zhang, Wen-Ting Gan, Wen-Ge Hao, Peng-Fei Wang, Zhuo-Yan Li, Lung-Ji Chang

**Affiliations:** ^1^Department of Pediatric Hematology/Oncology, Guangzhou Women and Children's Medical Center, Guangzhou, China; ^2^Shenzhen Geno-Immune Medical Institute, Shenzhen, China; ^3^Department of Molecular Genetics and Microbiology, College of Medicine, University of Florida, Gainesville, FL, United States

**Keywords:** secondary acute myeloid leukemia, myelodysplastic syndrome, myeloproliferative neoplasm, CAR T-cell therapy, CLL1

## Abstract

Secondary acute myeloid leukemia (sAML) is a high-risk AML evolving from heterogenous prior hematological disorders. Compared to de novo AML, sAML has even worse responses to current therapy and thus is associated with lower remission rates, inferior overall survival (OS) and higher relapse rates. Many efforts have been devoted to improving the overall but with limited success, and novel strategy is thus highly needed. Recent research has identified that CLL1 is highly expressed on AML leukemia stem cells and blasts cells but not on normal hematopoietic stem cells. In this case report, we treated a secondary AML patient with anti -CLL1 CAR-T therapy and achieved morphological, immunophenotypic and molecular complete remission for over 10 months. Although only one successful case is presented here, the anti-CLL1 CAR T-cells should be considered as another treatment option for secondary AML in the future.

## Background

The prognosis of patients with acute myeloid leukemia (AML) has been improving in the last decades through the intensification of combination chemotherapy and hematopoietic stem cell transplantation. However, the 5-year survival rate of patients with AML varies from 33.3–79.5% worldwide, as documented in the recent CONCORD-2 study (1), with a rate of only 40.1% in China.

Secondary AML (sAML) is considered high-risk as it evolves from heterogenous prior hematological disorders such as myelodysplastic syndrome (MDS), myeloproliferative neoplasm (MPN), and other malignancies exposed to cytotoxic agents or radiation therapy (2). Compared to *de novo* AML, sAML has worse response to current therapy, and thus is associated with lower remission rates, inferior overall survival (OS), and higher relapse rates (2). Many efforts have been devoted to improving the OS but with limited success; therefore, a novel strategy is highly needed (3).

Chimeric antigen receptor (CAR) T cells have emerged as a highly effective therapy for relapsed/refractory hematological malignancies (4–6); however, the efficacy of CAR T cells is unclear in AML. Recent research has shown that C-type lectin-like molecule-1 (CLL1) is highly expressed on AML leukemia stem cells (LSCs) and blast cells but not on the normal hematopoietic stem cells (HSCs) (7, 8), suggesting CLL1 as a promising target for novel AML therapy. Intriguingly, several groups have successfully developed CLL1-targeting strategies (9–13). These novel CLL1-directed therapies have shown some efficacy on AML cell lines *ex vivo*, primary human AML cells, and human AML patient-derived xenograft mice and monkey models *in vivo*; however, direct evidence regarding responses in patients with AML has not been reported.

In this case report, we treated a patient with secondary AML using anti-CLL1 CAR-T cells therapy and achieved morphological, immunophenotypic, and molecular complete remission for over a 10-month period. Although only one successful case is presented here, the anti-CLL1 CAR T-cells should be considered as another treatment option for secondary AML in the future.

## Case Presentation

**Figure 1:**
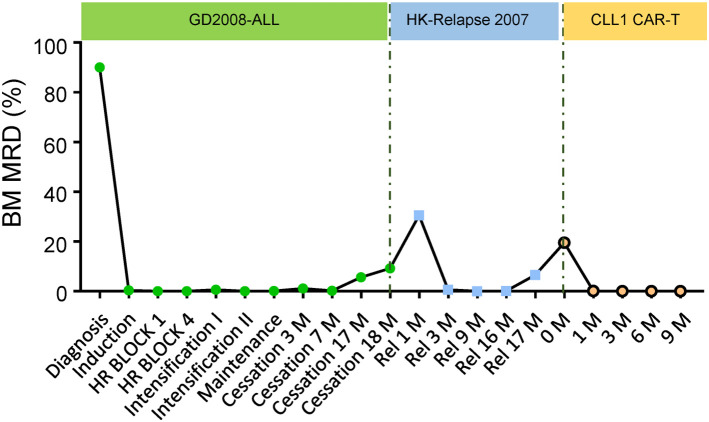
Therapeutic response for the anti-CLL1 CAR-T treatment. Plot of therapeutic response in this sAML during her primary diagnosis, 1st relapse and 2nd relapse including anti-CLL1 CAR-T cells therapy.

**Figure 2:**
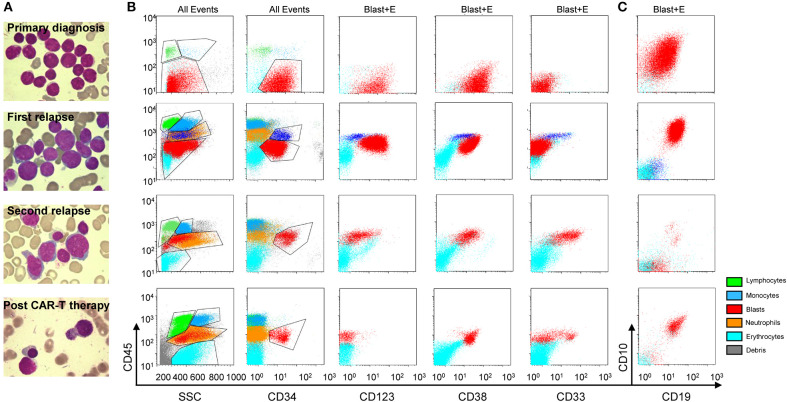
Morphologic and immunophenotypic evolution for this secondary AML case. Morphologic **(A)** (upper, primary diagnosis; upper middle, 1st relapse; lower middle, 2nd relapse; and lower, post anti-CLL1 CAR T-cell therapy) and flow cytometric **(B,C)** features at primary diagnosis (upper panel), 1st relapse (upper middle panel), 2nd relapse (lower middle panel), and post anti-CLL1 CAR T-cell therapy (lower panel) in this secondary AML case; gating: all events (All Events) or blast and erythroid cells (Blast+E). The green, blue, orange, light blue, gray and red dots represent lymphocytes, monocytes, neutrophils, erythrocytes, debris, and blasts population, respectively.

**Figure 3:**
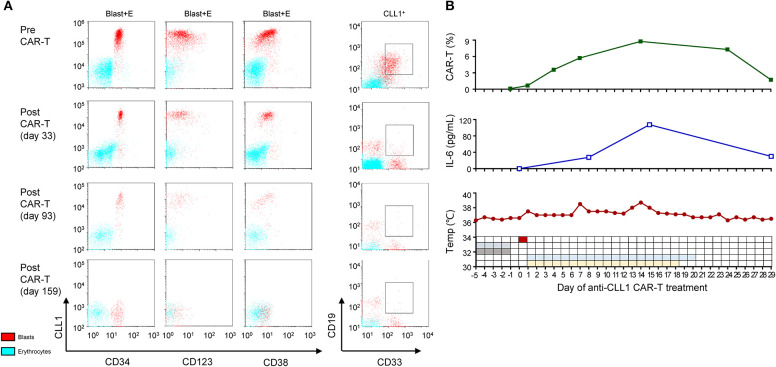
MRD and CRS status during acnti-CLL1 CAR T-cell therapy. **(A)** CLL1 population, intensity, and the blast percentage (gating on CLL^+^ cells) before (upper panel) and after (lower panel) anti-CLL1 CAR T-cell therapy. The blue and red dots represent erythrocytes and blasts population, respectively. **(B)** Anti-CLL1 CAR T-cells persistence (upper panel), serum IL-6 level (middle panel), and temperature and therapy scheme (low panel) during the first month of anti-CLL1 CAR T- cell therapy in this secondary AML case.

In early July 2018, a 10-year-old girl presented with pancytopenia and an elevated peripheral blood blast percentage while undergoing maintenance treatment for her first B-cell acute lymphoblastic leukemia (ALL) relapse. Bone marrow (BM) aspiration was performed every 2 weeks from July 2018, and blast percentage gradually increased from initial 7.5% to 19.5% within 1 month of observation (Figure 1). Furthermore, her medical history showed that she was diagnosed with high-risk B-ALL in October 2012, with a CD34^+^CD38^+^CD10^+^CD19^+^CD123^+^ immunophenotype. Initially, she was enrolled into the GD-2008-ALL clinical trial (NCT00846703) and then achieved complete remission (CR) after one course of induction therapy, while her minimal residual disease (MRD) evaluated by flow cytometry remained above 0.01% during all treatment phases (Figure 1). Unfortunately, she experienced an isolated BM relapse after a 1.5-year cessation of chemotherapy, and her relapse disease showed the same B-ALL immunophenotype as her first diagnosis. Due to the lack of economic support, she was treated with the HKPHOSG-Relapsed-ALL-2007 protocol and experienced persistent fever, pancytopenia, and elevated peripheral blood blast cells during the treatment (Figure 1). We then systemically compared samples from her primary diagnosis, along with her first and second instance of relapse. From this comparison, we found that the blast cells from the second relapse sample originated from the myeloid lineage (CD34^+^CD38^+^CD10^dim^CD19^dim^CD123^+^CD33^+^CLL1^+^) and not from the B cell lineage (Figures 2A–C). Moreover, we excluded the possibility of MDS-transformed AML because we did not identify a deletion of chromosome 5 or 7, or abnormal localization of immature precursors or marrow fibrosis. Molecular cytogenetic tests indicated the existence of MLL rearrangement, RUNX1^R204P^, and WT1^S381fs^ mutation (Supplementary Figure 1, Supplementary Table 1). Results were double confirmed by a peer review from the Shenzhen Gene-Immune Medical Institute (GIMI). Based on the 2017 European LeukemiaNet recommendations for AML, she was diagnosed as secondary AML and considered to be in the adverse risk group. Of note, CLL1 was highly expressed on the myeloid blast cells (Figure 3A, Supplementary Figure 2), which could be a target for CAR T-cell therapy.

Considering the long-term exposure to chemotherapeutic agents and the poor prognosis, her parents decided for her not to receive chemotherapy as a first-line therapy. With the help of GIMI, we developed a chimeric antigen receptor (CAR) containing a CLL1-specific single chain variable fragment, in combination with a fourth-generation CAR lentiviral vector (4SCAR) carrying T cell costimulatory signals for CD28-CD27-CD3z (14). The 4SCAR-CLL1 lentivector transfer efficiency for patient's PBMCs was ~95.06%. The patient received a lymphodepleting chemotherapy (cyclophosphamide 300 mg/m^2^/d and fludarabine 30 mg/m^2^/d for 4 days) before CAR T-cell transfer to enhance *in vivo* expansion of CAR T-cells, after which she received a single dose (5.8 × 10^7^ anti-CLL1 CAR-T cells, ~1.9 × 10^6^/kg) infusion over 10 min through a peripherally inserted central venous catheter on 11 September 2018. Subsequently, she experienced Grade I-II cytokine release syndrome (CRS) manifested by a temperature ≥38°C; at this time, she developed transient hypotension requiring fluid resuscitation under the CAR T-cell therapy management consensus guidelines (15, 16). Serum IL-6 level reached a peak on day 14 and then gradually decreased, which was consistent with the occurrence of CRS. Following the guidelines, glucocorticoids and tocilizumab intervention were not prescribed (Figure 3B). After the completion of CAR T-cell therapy, the patient achieved a morphological CR and was negative for MRD (<0.1%) on day 29. However, the CLL1^+^ cells were not completely eliminated until early in the 6 months after CAR T-cell therapy (Figure 3A, Supplementary Table 2). Upon complete remission, the BM response was monitored monthly during the first 3 months and every 3 months thereafter. Strikingly, the morphologic CR and MRD <0.1% were sustained for ~9 months at the time of this report's submission (Figures 1–3, Supplementary Table 2). However, we did not check CLL1 expression in peripheral blood samples in this case due to lack of experience. The persistence of anti-CLL1 CAR T-cells in peripheral blood was determined by quantitative real-time PCR, as previously described (17), during treatment and clinical follow-up. An effective expansion was achieved in the first month, which then dropped quickly thereafter; then, a low CAR T-cell level persistence was detected 5 months after CAR T-cell injection (Figure 3B). Even with the disappearance of CAR T-cells, a 10 month response (approximately) was achieved using one dose of anti-CLL1 CAR-T monotherapy in this patient, suggesting that anti-CLL1 CAR T-cells should be considered as an alternative strategy for AML or sAML in the future.

## Discussion

In this reported case, the main finding was the achievement of a surprisingly long-term complete remission with anti-CLL1 CAR T-cells therapy. Only grade I-II cytokine release syndrome was observed and successfully managed.

Anthracyclines- and cytarabine-based conventional chemotherapy are the main treatments for AML patients; however, both have significant toxicities. In addition, the overall prognosis for patients with this treatment has remained stagnant in the last two decades (18, 19). With the advent of next generation sequencing (NGS), more and more prognostically cytogenetic and molecular markers have been incorporated into AML risk classification and therapy (20–22). For example, tyrosine kinase inhibitors (FLT3-ITD inhibitor), IDH1 inhibitor ivosidenib, IDH2 inhibitor enasidenib, and BCL2 inhibitor venetoclax, monoclonal antibody-based therapy (anti-CD33 therapy); cellular therapy (CAR-T and TCR-engineered T-cell, and NK cell therapies), and novel regimens (decitabine and pracinostat) have been successfully developed (23–25). Although new targeted agents have been extensively introduced into clinical treatment, relapse still remains the most significant issue influencing the survival of patients with AML. Allogeneic hematopoietic stem cell transplantation remains the last hope for these patients, indicating the important role of normal immune reconstitution for successfully treating patients with AML.

In contrast to chemotherapy and new FDA-approved targeting agents, cellular CAR-T cell therapy and cytotoxic T lymphocytes can minimize systemic cytotoxicity and morbidity while generating maximal anti-tumor activity (22). However, in this study, we did observe low anti-CLL1 CAR T-cells expansion during the CAR T-cell therapy. The selection of CD28/CD27 costimulatory signals might be the reason for short-term persistence. CD28 and 4-1-BB are two well-established costimulatory signals for the generation of CAR-T cells. CD28 costimulation can augment T-cell receptor (TCR) signaling and increase TCR sensitivity, which is very helpful in facilitating T-cell responses against weak agonist peptides. Compared to CD28, 4-1-BB costimulation can enhance T-cell expansion and maintenance and rescue T-cells from anergy and exhaustion. The low effective CAR-T cell expansion and the lack of long-term persistence might be the bottleneck in the future clinical application of this therapy.

In conclusion, our prolonged survival observation in this patient with sAML showed that anti-CLL1 CAR-T might be of great potential in AML treatment. Interestingly, Liu F et al. has reported the efficacy of combination CLL1-CD33 CAR-T cells in a 6-year-old girl with sAML transformed from prior faconi anemia and juvenile myelomonocytic leukemia (22). Given that B-cell aplasia is an indicator of CD19 CAR T-cell therapy, frequencies of CLL1 expressing mononuclear cells might be used as an indicator in a similar way, to monitor the efficacy of CAR T-cells. As mentioned above, the persistence of CLL1 expressing mononuclear cells might serve as an indicator of anti-CLL1 CAR-T efficacy and should be included to monitor the anti-CLL1 CAR-T in the future. To our knowledge, this is the second successful case of anti-CLL1 CAR-T application for secondary AML treatment, highlighting its promising potential in the high-risk AML population.

## Ethics Statement

The studies involving human participants were reviewed and approved by GIMI-IRB-16001. Written informed consent to participate in this study was provided by the participants' legal guardian/next of kin. Written informed consent was obtained from the individual(s), and minor(s)' legal guardian/next of kin, for the publication of any potentially identifiable images or data included in this article.

## Author Contributions

HZ, W-TG, and L-JC performed the research, analyzed the data, and wrote the paper. W-GH and P-FW included the patients and collected clinical data. Z-YL contributed to clinical nursing. All authors critically revised the manuscript and approved the final version.

## Conflict of Interest

The authors declare that the research was conducted in the absence of any commercial or financial relationships that could be construed as a potential conflict of interest.

## Supplementary Material

Supplementary Figure 1 MLL-rearrangement detection in this secondary AML patient. Interphase FISH using the LSI MLL dual color, break-apart rearrangement probe set. Interphase nucleus harboring the MLL translocation, 1 fusion signal (yellow), and 2 separate signals (red and green).

Supplementary Figure 2 The isotype control for flow cytometry. The gray shaded area represented isotype controls for multi-color flow cytometry.

Supplementary Table 1 Immunophenotypic, cytogenetics and mutation features of this patients.

Supplementary Table 2 Clinical follow-up during anti-CLL1 CAR-T therapy in this secondary AML case.
