# Supplementary figures and images for: Successful Anti-CLL1 CAR T-Cell Therapy in Secondary Acute Myeloid Leukemia
Source: Front Oncol. 2020 May 27;10:685. doi: 10.3389/fonc.2020.00685 (PMC7266936; doi:10.3389/fonc.2020.00685)

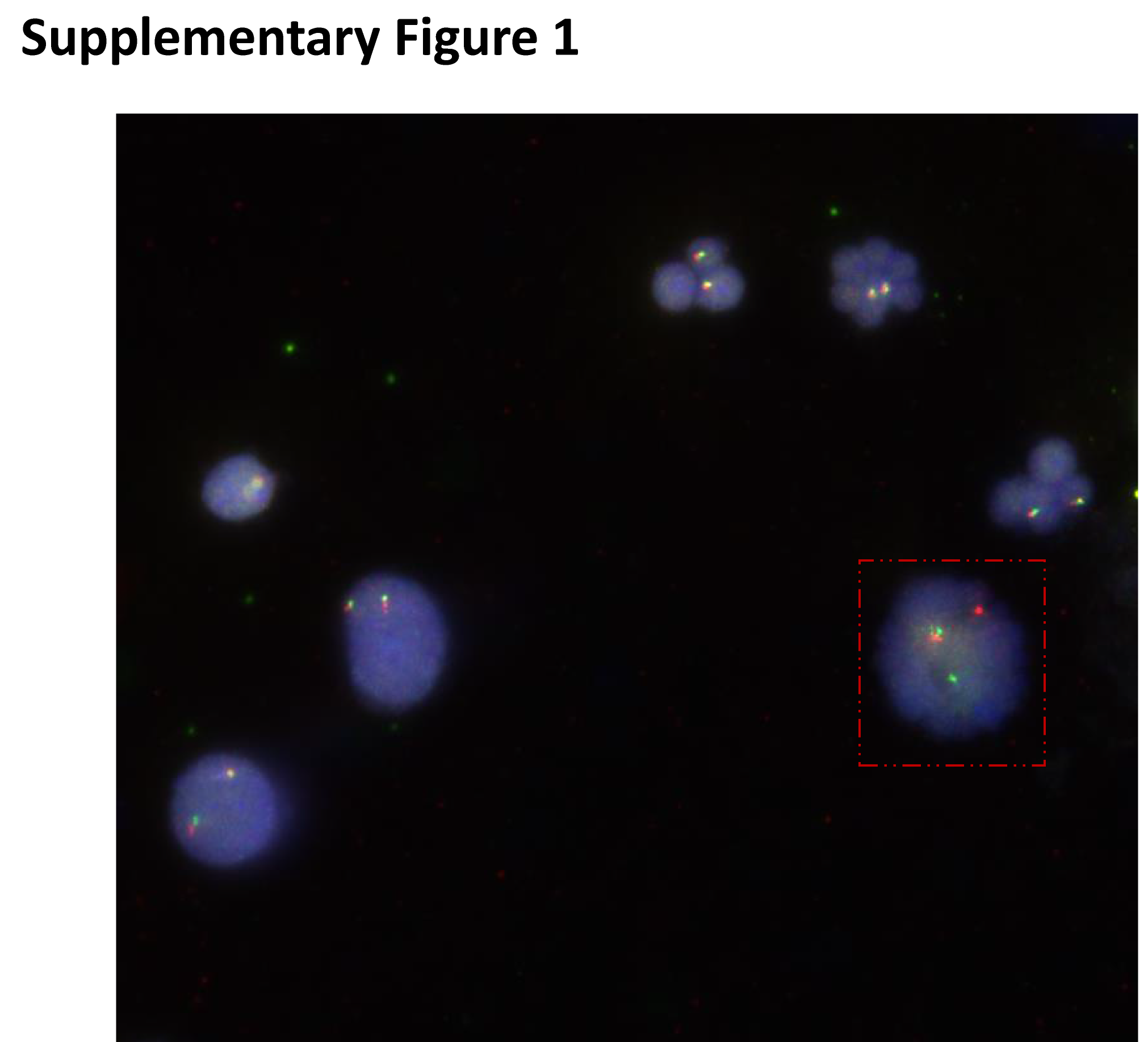

Supplement: Supplementary Figure 1 — MLL-rearrangement detection in this secondary AML patient. Interphase FISH using the LSI MLL dual color, break-apart rearrangement probe set. Interphase nucleus harboring the MLL translocation, 1 fusion signal (yellow), and 2 separate signals (red and green). [file Image_1.TIF]

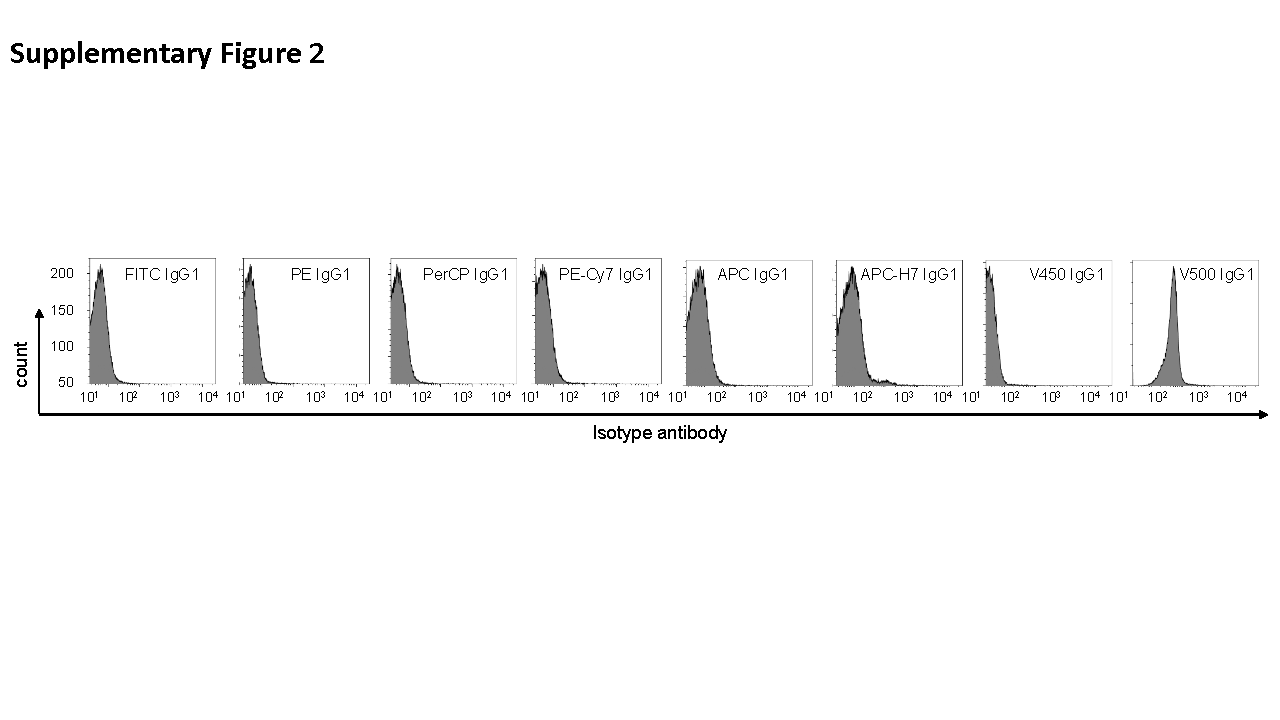

Supplement: Supplementary Figure 2 — The isotype control for flow cytometry. The gray shaded area represented isotype controls for multi-color flow cytometry. [file Image_2.TIFF]
